# A Preliminary Study of Pre-Season Taekwondo Preparation Strategy: Personal Isolation Training Effect for Elite Athletes

**DOI:** 10.3390/ijerph182010570

**Published:** 2021-10-09

**Authors:** Yu-Chi Sung, Yi-Zhen Yang, Che-Chia Chang, Chun-Chung Chou

**Affiliations:** 1 Department of Combat Sport and Martial Arts, Chinese Culture University, Taipei 11114, Taiwan; idcord2@gmail.com; 2 Department of Physical Education, University of Taipei, Taipei 10048, Taiwan; ok131055@gmail.com; 3 Sports Administrative Organization, Center of General Education, National Chi Nan University, Nantou 54561, Taiwan; cocky61055105@gmail.com; 4 Physical Education Office, National Taipei University of Technology, Taipei 10608, Taiwan

**Keywords:** detraining, physical activity volume, taekwondo specific training

## Abstract

Background: The global coronavirus disease pandemic (COVID-19) has had a considerable impact on athletic competition and team sports training. Athletes have been forced to train alone at home. However, the isolation training model effects are still unknown. Purpose: This study compared the effects of personal isolation training (PIT) and detraining (DT) on specific sport performances (flexibility, power, reaction time, acceleration, and aerobic capacity) and body composition in elite taekwondo athletes. Methods: Eleven elite taekwondo athletes were recruited as voluntary subjects. Athletes were randomly paired by weight into the personal isolation training group (PIT group: *N* = 5, age: 21.2 ± 0.4 years, BMI: 22.2 ± 0.8 kg/m^2^) or detraining group (DT group: *N* = 6, age: 19.8 ± 0.3 years, BMI: 23.1 ± 1.0 kg/m^2^). All subjects performed the same training content prior to the pre-test (T1). When the pre-test was completed, all subjects underwent 12 weeks of PIT or DT. Athletes were then administrated the post-test (T2). The athlete’s sport performances and body composition were measured to compare the differences between the two groups (PIT and DT) and two phases (T1 and T2). Results: There were no significant differences in kicking reaction time and flexibility in both groups (*p* > 0.05). The PIT showed significant improvements in 10 m (10M) sprint performance (*p* < 0.05), and displayed a progress trend in Abalakov jump performance. In addition, the PIT resulted in a better change response in 10M sprint performance (PIT: −4.2%, DT: +2.1%), aerobic endurance performance (PIT: −10.2%, DT: −18.4%), right arm muscle mass (PIT: +2.9%, DT: −3.8%), and trunk muscle mass (PIT: +2.2%, DT: −1.9%) than DT (*p* < 0.05). The fat mass percentage showed a negative change from T1 to T2 in both groups (*p* < 0.05). Conclusions: PIT showed a trend toward better body composition (arm and trunk muscle) and sport performances (10M sprint and aerobic capacity) compared to DT. This finding may provide information on the effectiveness of a personal isolation training model for optimal preparation for taekwondo athletes and coaches. It may also serve as a useful and safe guideline for training recommendations during the coronavirus disease (COVID-19).

## 1. Introduction

Taekwondo is a high-impact, contact sport with athletes requiring an intense training program to develop specific physical techniques to perform best and reduce the risk of injury [1,2]. These taekwondo physical attributes need to be developed through a well-planned, regular training program [2]. However, the spread of coronavirus disease (COVID-19) has led to a global pandemic from 2020 to the present. As a result, society has adopted many isolation steps that have had a considerable impact on athletic competition and team sports training. The most important requirement for pandemic control is human isolation, which forces athletes to train alone at home. The consequences of isolation are: lack of structured training and competition, lack of realistic practice space and impact, lack of communication between athletes and coaches, and in severe cases, suspension/interruption of training. Due to the training interruption caused by COVID-19, this process is similar to the traditional detraining/training cessation model [3]. The term detraining refers to the partial or complete loss of prior anatomical, physiological, and functional adaptations due to interruptions or cessation in exercise training [4]. Although the potential impact of the COVID-19 pandemic is unprecedented in scale, there are examples of the consequences of retraining. For example, soft tissue injuries were more frequent when players experienced a 20 week detraining in the National Football League in 2011 [5].

Previous studies showed that detraining has a negative effect on the body composition and overall inflammatory state of taekwondo athletes, as well as a decline in aerobic capacity [6,7]. Although previous studies on training cessation showed positive effects on psychological recovery and injury recovery [8,9], the effects of detraining caused by canceling and suspending the training program might cause considerable stress, frustration, anxiety, and sadness for elite athletes [10,11]. In addition, training cessation has produced decreases in strength, force, and power. This effect was found according to the training cessation duration, age, and training status [12]. During short training cessation periods (no longer than three weeks), athletes can still maintain training-specific adaptations. However, athletes experiencing long training cessation periods (six to eight weeks) show a loss in exercise performance adaptations and optimal body composition status [6,13,14].

Recent data on taekwondo training show that training in the taekwondo arena has a positive effect on improving the aerobic capacity of taekwondo athletes [15]. However, when external factors (e.g., the prevalence of COVID-19) cause the isolation of people from taekwondo group training, the change to personal isolation training will have different results on the body composition and performance in elite taekwondo athletes. This is a practical issue that should be explored as COVID-19 has now spread worldwide. In addition, a recent study suggested that during the dissemination phase of COVID-19, strategies should be planned and structured to ensure that athletes maintain a level of intensive training [16]. Therefore, the effectiveness of personal isolation training (PIT) and the research evidence are worth investigating. Although PIT is somewhat difficult to implement, it may be a practical strategy in situations when group exercises are not possible. We assumed that PIT will be better than training cessation in maintenance fitness level and body composition. This study compared the effects of PIT and detraining (DT) on specific sport performances (flexibility, power, reaction time, acceleration, and aerobic capacity) and body composition in elite taekwondo athletes.

## 2. Materials and Methods

### 2.1. Participants

Eleven elite taekwondo athletes (8 males and 3 females) volunteered for this study. These volunteers had at least 8 years of competitive experience in the sport and all of these athletes placed in the top 3 at national competitions within the past 2 years. All participants were randomly paired by weight into the personal isolation training group (PIT group: *N* = 5, age: 21.2 ± 0.4 years, BMI: 22.2 ± 0.8 kg/m^2^) or detraining group (DT group: *N* = 6, age: 19.8 ± 0.3 years, BMI: 23.1 ± 1.0 kg/m^2^). There were no significant differences in anthropological parameters between the two groups (weight, BMI, lean body mass, body fat percentage, and muscle mass; *p* > 0.05). All participants were confirmed to be free of any specific diseases and had no skeletal or muscular injuries within the last three months. To ensure good dietary pattern control throughout the 12 week period, we provided detailed nutritional guidelines to all athletes [13]. This study was approved in accordance with the recommendations of the Institute Review Board (IRB) of the Taipei Medical University. All subjects were voluntary and completed written informed consent after they understood the whole procedure and experimental risks.

All athletes performed the same training content prior to the pre-test (T1) (10 training sessions per week; 14–15 h per week, including 2–2.5 h of aerobic training, 7.5–8 h of taekwondo-specific skill training, 2.5 h of strength and conditioning training, and 2 h of stretching and flexibility training).

**Figure 1:**
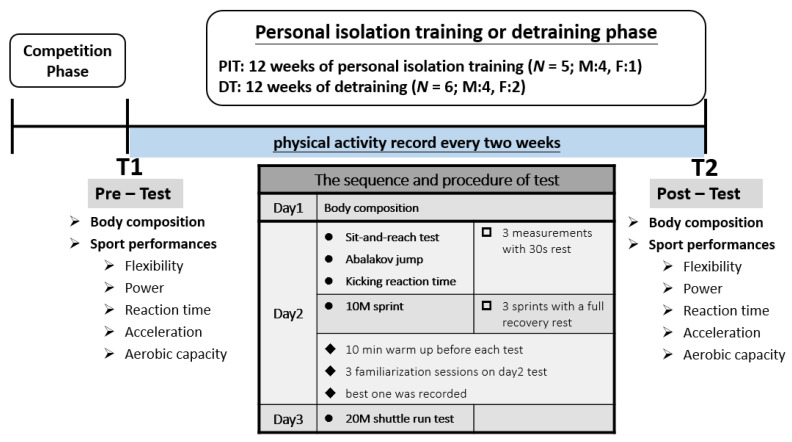
Experimental design time line.

When the pre-test was finished, all subjects underwent 12 weeks of PIT or DT for the post-test (T2). The same procedure and sequence was used in T1 and T2, which consisted of sport performances and body composition. In order to minimize the possible confounding factors from the previous performance test, we arranged the measurement sequence according to the energy- and difficulty-demands of exercise performance tests. In brief, the T1 and T2 sequence was body composition on the first day with the sit-and-reach test (flexibility), Abalakov jump (power), and kicking reaction time (reaction time); a 10 m sprint (acceleration) on the second day; and a 20 m shuttle run (aerobic capacity) on the third day. Each test was performed at 08:00–11:00 AM. Moreover, there were 4 tests performed on Day 2, and each test was separated by a standard interval for at least 30 min to ensure sufficient recovery. The body composition test included weight, BMI (body mass index), lean body mass, body fat weight, body fat percentage, right arm, left arm, trunk, right leg, and left leg. In both groups, physical activity was recorded using a 3 day physical activity scale (every 2 weeks) during the 12 week period. The procedure is shown in Figure 1.

### 2.2. Physical Activity Evaluation and Personal Isolation Training/Detraining

The physical activity was recorded using the Bouchard three-day physical activity record [17], and the validity and the reliability of the questionnaire was demonstrated [18]. During the 12 weeks of PIT or DT, the physical activity was recorded every 2 weeks. All participants recorded three days of activity on a daily basis to assess the energy consumed by the participant’s daily physical activity. The activity record table divides the day into 96 intervals every 15 min. Subjects must record the physical activity items they remember in the interval (from 1 to 9) according to the time. The researcher multiplies the total number of daily categorical values for each day on the record table using the energy consumption value represented by kcal/kg/day, which represents the energy expenditure per kilogram of body activity per day value (in kcal/kg/day).

Subjects in the DT group did not perform any taekwondo or physical training during the 12 week detraining period.

Subjects in the PIT group were trained five times per week during the 12 week period. The training content was: taekwondo-specific basic skills training, strength and conditioning training, and stretching and flexibility training. The PIT and DT training contents are shown in Table 1.

### 2.3. Sport Performances

The sport performances tests in the pre-test (T1) and post-test (T2) had the same procedure and sequence. These tests were finished in two days in which the sit-and-reach test, Abalakov jump, kicking reaction time, and the 10M sprint tests were performed on one day and the 20 m shuttle run test was performed on another day. There were 10 m warm-up and familiarization sessions before each test. The best test result was recorded. The intraclass correlation coefficient (ICC) for the day 2 test results were above 0.83.

#### 2.3.1. Flexibility Test

The lower limb flexibility was assessed by the sit-and-reach test, which was performed according to the previous study [20]. The participant sat on the floor with both legs extended. With one hand on top of the other, the participant slowly reached forward as far as possible and held that position for 2 s. The test was repeated three times. The best one was recorded.

#### 2.3.2. Abalakov Jump (AJ)

**Figure 2:**
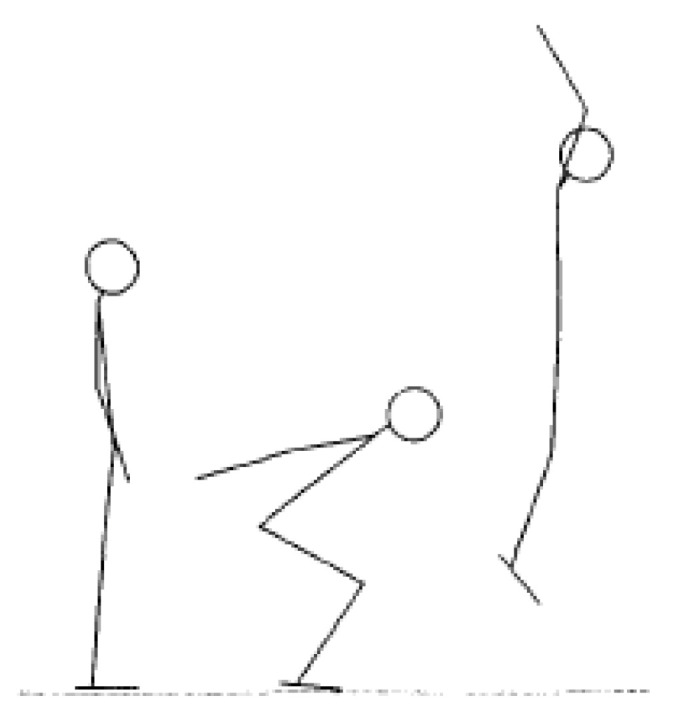
Sequences of Abalakov jump.

The Abalakov jump test was performed according to the previous study [21]. Participants were asked to perform three jump practices for familiarization before the real test. In the formal experiment, participants performed three jumps and were asked to jump as high as they could in the vertical direction, with a 30 s rest period between each jump. The best jump was recorded. The starting position was upright with the arms at the sides, and the arms were swung backward as their body went down with the knees bent. The athlete then swung their arms in a downward, forward, and upward stance for the vertical jump (Figure 2). All jump measurements were performed on a force platform (OR 6-WP-1000, Advanced Mechanical Technology, Inc., Watertown, MA, USA). The data were collected at a sampling rate of 1000 Hz. Kinematic and kinetic data were recorded using Cortex software (Version 3.6, Motion Analysis Corporation, Santa Rosa, CA, USA). The Abalakov jump test results were presented in watt units and for further analysis.

#### 2.3.3. Kicking Reaction Time

**Figure 3:**
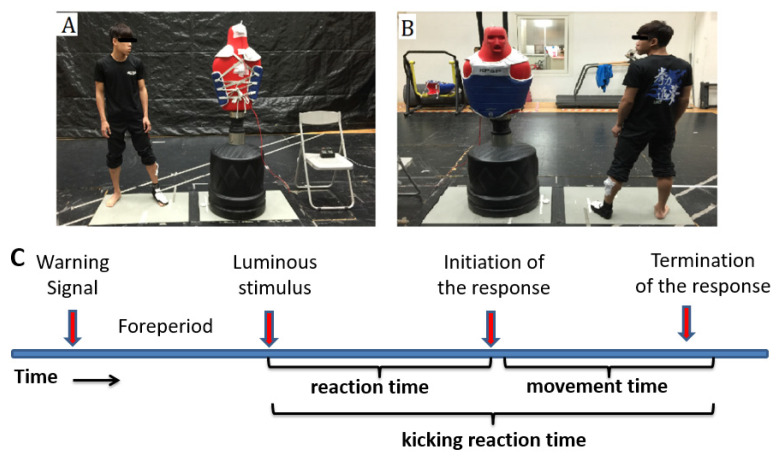
The kicking reaction time test venue layout (**A**,**B**) and the response time theory (**C**).

Participants were asked to use the front roundhouse kick using the dominant leg in the kicking reaction time test. The participant’s trochanteric height (i.e., the full length of the trochanter to the ground) was measured to determine the distance to the kicking target (the distance was used for each test), and the length from the half-point of the fibular head to the middle of the lateral malleolus would be measured to set the accelerometer sensor (DTS 3D Accelerometer, Noraxon U.S.A. Inc., Scottsdale, AZ, USA). Participants were instructed to put on an electronic foot sock to determine the effective and valid kick. The target wore a hogu connected to a laptop to show whether the kick was valid. An electronic device was set on top of the target emitting a luminous stimulus indicating the moment the athlete kicked the target. Another accelerometer sensor was set at the same position for recording. The time spent between the emitted stimulus and the foot movement was considered as the reaction time. The time spent between the foot movement and the target being kicked was considered as the movement time. The reaction time and the movement time were added together as the kicking reaction time. In the formal experiment, participants performed three kicks, with a 30 s rest period between each kick. The best kick was recorded. The kicking reaction time test procedure is shown in Figure 3.

#### 2.3.4. 10 m (10M) Sprint Test

**Figure 4:**
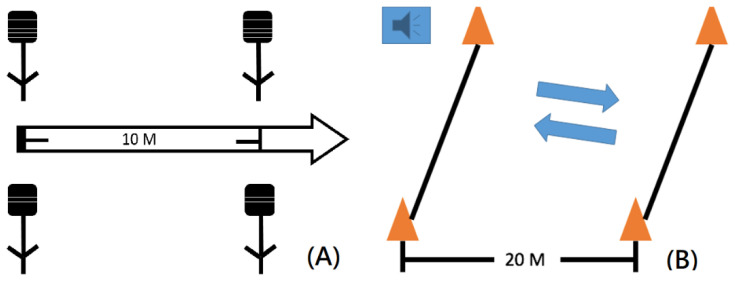
The field layout of (**A**) 10 m sprint, and (**B**) 20 m shuttle run.

The 10M sprint test was performed according to the previous study [22]. Participants were asked to complete the sprint at maximal speed, with three familiarization sessions and a 10 m warm-up before the test began. The 10M sprint test was conducted three times, with full rest between each test, and the best results was recorded. The 10M sprint test time was assessed using infrared timing gates (Smart Speed, Fusion Sport, Queensland, Australia), which were positioned at the start line and at 10 m at a height of approximately 1.0 m. At the starting line, the athlete took a two-point (upright) starting position with the feet staggered and the preferred foot forward. The recording time was started when the laser at the starting gate was broken. The recording was completed when the participant passed the second gate (Figure 4A).

#### 2.3.5. Aerobic Capacity Test

A 20 m shuttle run test introduced by Leger and Mercier was used to determine the participant’s maximal aerobic capacity. The 20 m shuttle run test contained interval exercise components and is one of the field-tests that is suitable for combat-type sports to determine aerobic capacity [23]. The test required every participant to run between 2 lines spaced 20 m apart while keeping pace with a sound signal (Figure 4B). The sound signal frequency started at 8.5 km/h and increased 0.5 km/h each minute. Participants were expected to run using the sound signal until exhaustion, at which point the final level was used to calculate the maximum oxygen uptake. Aerobic capacity could be estimated using the following equation: (VO_2_max; mL/kg/min) = 31.025 + 3.238 × (maximal speed (km/h), determined by level complete) − 3.248 × Age (yrs) + 0.1536 × speed (km/h) × Age (yrs) [24].

### 2.4. Body Composition

The bioimpedance body composition analyzer (InBody 720, Biospace Co., Ltd., Seoul, Korea) was used to measure the body mass (BM), lean body mass (LBM), skeletal muscle (SM), and body fat percentage (% BF). The measurement was taken after an overnight fast (12 h) and between 08:00 and 09:00 AM [7].

### 2.5. Statistical Analysis

Data were presented as mean ± standard error of mean (Mean ± S.E.M.), and the percent changes (Δ% between pre- and post-test) in the measured parameters were calculated using the formula = [(post-test value − pre-test value)/(pre-test value)] × 100%. Statistical analysis was performed using SPSS statistical analysis software (Version 23, IBM, Armonk, NY, USA). Data were analyzed using two-way repeated measure ANOVA. The effect size was calculated by Cohen’s f for ANOVA and Hedges’ g for percent changes. The post hoc tests were used to analyze the differences when significance was found. The alpha values of statistically significant for all comparisons were set to 0.05.

## 3. Results

### 3.1. Sport Performances

**Figure 5:**
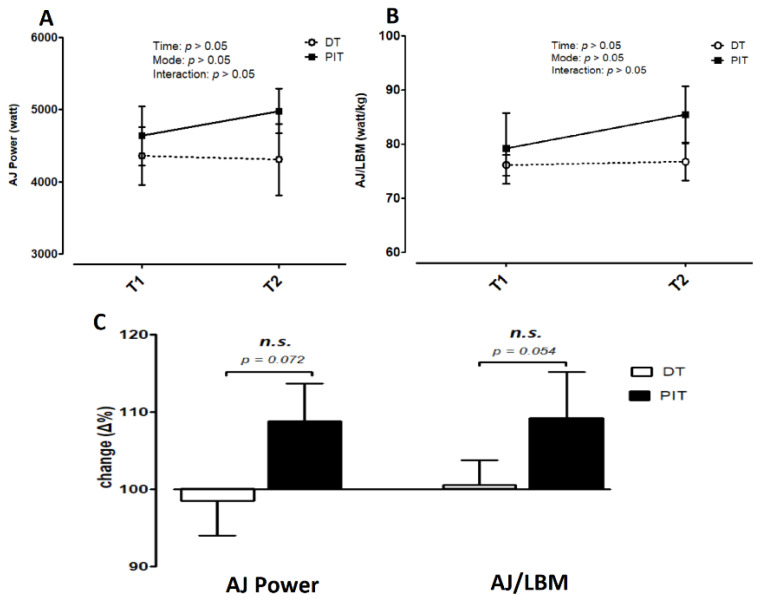
Abalakov jump (AJ) and AJ/lean body mass (LBM). (**A**) AJ and (**B**) AJ/LBM were measured in pre-test (T1) and post-test (T2). The rates of AJ and AJ/LBM change from T1 to T2 are displayed (**C**). Data are expressed as mean ± S.E.M.

Figure 5A,B display the AJ and AJ/LBM results for both groups in T1 and T2, respectively. For the AJ, there was no significant interaction effect (*F* = 1.568, *p >* 0.05, *η*^2^ = 0.148). The main effects for time (*F* = 1.197, *p >* 0.05, *η*^2^ = 0.117) or mode (*F* = 0.282, *p >* 0.05, *η*^2^ = 0.030) were not significant in AJ (Figure 5A). For the AJ/LBM, there was no significant interaction effect (*F* = 1.673, *p >* 0.05, *η*^2^ = 0.157). There were no significant main effects for time (*F* = 2.549, *p >* 0.05, *η*^2^ = 0.221) or mode (*F* = 1.017, *p >* 0.05, *η*^2^ = 0.102) in AJ/LBM (Figure 5B). Likewise, after calculating the AJ and AJ/LBM rate of change from T1 to T2, the PIT group showed an increase trend of 9% in AJ and AJ/LBM, whereas the DT group showed a decrease trend of 2%. Regarding the rates of change comparison, there was no difference between the two groups in AJ (*p* = 0.072, ES = 0.97) and AJ/LBM (*p* = 0.054, ES = 0.81) (Figure 5C).

**Figure 6:**
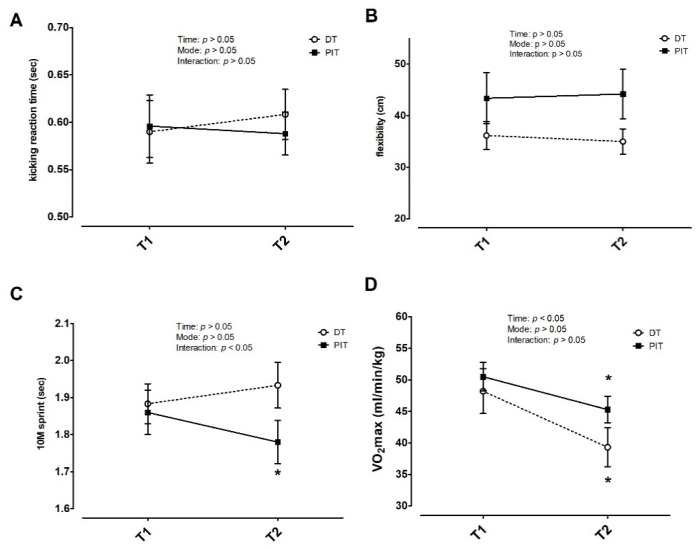
Kicking reaction time results (**A**), flexibility (**B**), 10M sprint (**C**), and VO_2_max (**D**) in T1 and T2. * *p* < 0.05 vs. T1 under the same condition. Data are expressed as mean ± S.E.M.

For the kicking reaction time test, no significant interaction effect was found (*F* = 0.948, *p >* 0.05, *η*^2^ = 0.095). The main effects for time (*F* = 0.146, *p >* 0.05, *η*^2^ = 0.016) or mode (*F* = 0.033, *p >* 0.05, *η*^2^ = 0.004) were not significant in kicking reaction time (Figure 6A). For the flexibility, there was no significant interaction effect (*F* = 0.934, *p >* 0.05, *η*^2^ = 0.094). There were no significant main effects for time (*F* = 0.32, *p >* 0.05, *η*^2^ = 0.004) or mode (*F* = 2.553, *p >* 0.05, *η*^2^ = 0.221) in flexibility (Figure 6B). For the 10M sprint, a significant interaction effect (*F* = 16.609, *p* < 0.05, *η*^2^ = 0.649) was observed. Post hoc analyses revealed that the 10M sprint result was significantly better in T2 than T1 of the PIT group (*p* = 0.002) (Figure 6C). Likewise, after calculating the 10M sprint change rate from T1 to T2 (PIT: −4.2%, DT: +2.1%), there was a significant difference between the two groups (*p* = 0.001, ES = 2.66). For the aerobic capacity test result, the interaction effect (*F* = 4.179, *p >* 0.05, *η*^2^ = 0.317) and mode effect (*F* = 4.179, *p >* 0.05, *η*^2^ = 0.317) were not significant. A significant main effect for time (*F* = 61.041, *p <* 0.05, *η*^2^ = 0.872) was observed in VO_2_max (Figure 6D). Post hoc analyses revealed that there were significant decreases in T2 compared with T1 for both groups (PIT: −10.2%, DT: −18.4%, *p* < 0.05), and the DT had a greater decline from T1 to T2 than PIT did (*p* = 0.013, ES = 1.62) (Figure 6D).

### 3.2. Body Composition

The weight, BMI, LBM, left arm muscle mass, trunk muscle mass, right leg muscle mass, and left leg muscle mass did not show any significant differences during 12 weeks of PIT or DT, and there were no differences between the two groups. For fat mass percentage, there were no significant interaction effect (*F* = 0.841, *p >* 0.05, *η*^2^ = 0.085) and mode main effect (*F* = 1.273, *p >* 0.05, *η*^2^ = 0.124). A significant main effect for time (*F* = 9.895, *p <* 0.05, *η*^2^ = 0.524) was observed in fat mass percentage. Post hoc analyses revealed that there was a significant increase from T1 to T2 for both groups (*p* < 0.05). For right arm muscle mass, a significant interaction effect (*F* = 5.295, *p* < 0.05, *η*^2^ = 0.370) was observed. Post hoc analyses revealed that the right arm muscle mass result showed a significant decrease from T1 to T2 of the DT group (*p* < 0.05). Likewise, after calculating the rate of body composition change from T1 to T2, there were significant differences between the two groups in right arm muscle mass (*p* = 0.025, ES = 1.39) and trunk muscle mass (*p* = 0.038, ES = 1.21). The PIT and DT body composition data are shown in Table 2.

### 3.3. Physical Activity

**Figure 7:**
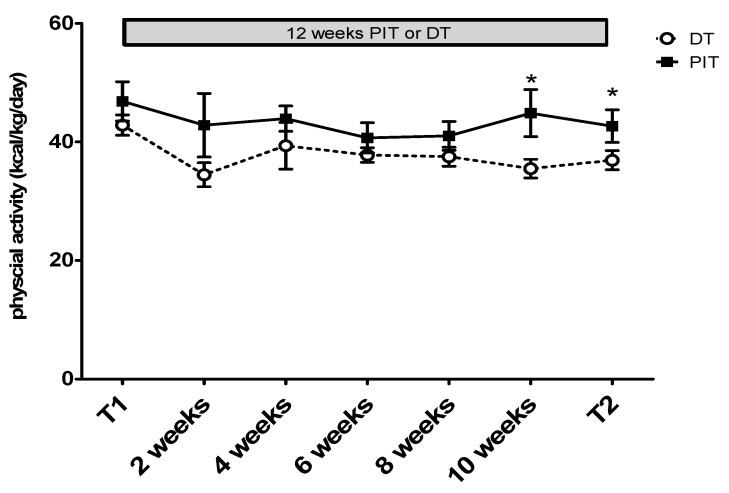
Physical activity volume results. * *p* < 0.05 vs. DT at the same time-point.

For physical activity, the PIT group (42.7 ± 2.8 kcal/kg/day) demonstrated a significantly higher average volume (*p* = 0.047, ES = 1.13) than the DT group did (36.9 ± 1.6 kcal/kg/day) (Figure 7).

## 4. Discussion

This study evaluated the off-season personal isolation training and detraining effects on sport performances and body composition variables in elite taekwondo athletes. It is the first research to compare the effects of 12 week personal isolation training and training cessation for Taekwondo athletes. The main finding of this study was that during the 12 week personal isolation phase, the training group showed significant improvements in 10 M sprint performance and displayed a progress trend in Abalakov jump performance. In addition, the personal isolation training mode resulted in a better change response in the 10 M sprint, aerobic endurance performance, right arm muscle mass, and trunk muscle mass than detraining did. The fat mass percentage and aerobic capacity showed a negative change from T1 to T2 in both groups; however, the detraining mode induced a greater decline from T1 to T2 than the personal isolation training mode did. These findings provide information about the effectiveness of the personal isolation training model for the best preparation for taekwondo competitors.

We found different trends in the sport performances between the personal isolation training group and the detraining group. The data from this study suggested that even if the personal isolation training model was used during the off-season, it is beneficial for taekwondo athletes to maintain their performance in the off-season as long as the training is continued. However, the personal isolation training mode used in this study is to some extent similar to that of reduced training. In a previous study on top-level kayakers, the effects of 5 weeks of reduced training and complete training cessation on physiological parameters were compared. The two strategies were found to lead to a reduction in maximal oxygen uptake, but the reduction in reduced training was less than that in training cessation, which is consistent with the findings of our study. This study found that the maximum oxygen uptake in the reduced training group was reduced by ~10%, which was less than that in the training cessation group (~18%) [25]. Our results are also consistent with the findings in other exercise model athletes, e.g., in well-trained endurance athletes [26,27], soccer players [28], and team players [29], the maximal oxygen uptake decreased significantly after 2–8 weeks of reduced training. In addition, a recent study found that taekwondo training is effective in improving VO_2_max [15], and it is possible that the lack of taekwondo combat training might have a negative impact on aerobic endurance performance. In this study, we demonstrated that the personal isolation training mode resulted in less decline in aerobic endurance performance than detraining (PIT: −10.2%, DT: −18.4%) after a 12 week period (Figure 6D). Moreover, the greater maximal oxygen uptake reduced after detraining, which would result in a higher loss in previous training adaption, and would have more obvious negative effects on cardiopulmonary fitness, the energy metabolism system, and endurance exercise performance [4,30].

Our study found that after 12 weeks of personal isolation training, taekwondo elite athletes had a significant improvement in their 10 m short distance sprint, and the PIT mode showed more improvement than DT did in the 10M sprint change% from T1 to T2 (PIT: −4.2%, DT: +2.1%) (Figure 6C). However, after 12 weeks of training cessation, the above-mentioned specific exercise indicators decreased significantly, which is consistent with the results from a previous study [31]. Some of the previous studies showed a degree of decrease in sprinting performance and a small or moderate decrease in muscle strength [4,9,13], whereas some studies found no negative effects on sprinting performance under reduced training or training cessation [14,32,33,34,35,36,37]. We suggest that such differences might be influenced by changes in training volume and intensity, as well as the length of the reduced training or training cessation period. In addition, the PIT group still maintained ~30% of the training amount, and the training intensity was the same as the competitive phase, which might be beneficial to sport performances maintenance, and might provide better explosive strength and sprinting ability development, which could be a better start for taekwondo athletes at the beginning of the new season. Although there were no significant differences between the two groups in AJ performance, the PIT group showed an increase trend of 9% in AJ and AJ/LBM, whereas the DT group showed a decrease trend of 2%. However, at the cessation of training, the volume and intensity in the detraining group led to a negative decrease in sport performance, which is consistent with previous studies [4,30]. Consistent with our findings, a previous study reported that taekwondo athletes showed a significant decrease in muscle strength and anaerobic power, but no significant change in reaction time during three weeks of detraining [38]. In this study, the kicking reaction time showed no significant differences in either group; we speculated that the participants in this study were elite taekwondo athletes and that the reaction time would be difficult to change during detraining. Moreover, the negative decline changes in detraining effect in flexibility were also confirmed by a previous study [39]. Although no negative or positive changes in flexibility were observed in either group in this study, we concluded that 0.5 h of stretching and flexibility training per week did not cause a significant improvement in PIT.

In the body composition analysis, we found that PIT during the off-season could help elite taekwondo athletes maintain their upper limb and trunk muscle mass. In the physical activity volume, we found that the average physical activity volumes for the PIT and DT groups were 42.7 (kcal/kg/day) and 36.9 (kcal/kg/day), respectively, and the PIT group was significantly higher than the DT group in average physical activity volume (*p* < 0.05). The difference in physical activity volume between the two groups was caused by the presence or absence of training, as the PIT group continued training for approximately 7 h per week during the 12 week period. We speculated that the maintenance of training was the main factor causing the difference in body composition between the two groups. In addition, for taekwondo kicking, the main function of the trunk is to support and maintain body balance stability and control of the stagnant foot during the kick rotational force [40]. Moreover, we also found that, after 12 weeks, the detraining model increased body fat significantly. This is consistent with the results of previous studies [6], which showed that taekwondo athletes gained 21.3% in body fat after detraining for 8 weeks. As a result of the spread of COVID-19, restrictions on physical activity have led to a significant increase in weight among international-level kickboxers, and a large part of the weight gain in terms of our inferences is caused by increased fat [41]. There are a few studies that are consistent with our current finding. In a study on elite young football players, it was found that after four weeks of detraining, the percentage of body weight and body fat nearly increased, but the proportion of increase was not significant [42], and the results were in line with our present findings with elite taekwondo players [43]. However, not all studies exhibited consistent findings. In a study of 14 world-class Kayaking athletes, it was found that after five weeks of reduced training or cessation of training, the bodyweight of athletes in the reduced training group remained unchanged, but decreased significantly by 3% in the stop training group [26]. In addition, that study found that the two-week cessation of training for excellent football players in the off-season did not make significant changes in body composition [9]. Therefore, we further speculate that these inconsistent results may be due to the inconsistent or shorter time of period for cessation or reduced training.

There are some limitations in this study. A major consideration when taekwondo athletes train under PIT is the setting of training intensity and volume. Due to the lack of proper space, it is difficult for coaches to monitor training intensity and ensure that the training used by athletes at home is appropriate to maintain fitness and performance at the desired level. Taekwondo is a combat sport and the impact during training and the feedback of impact are very important for taekwondo athletes, and this is lacking in the PIT model. As elite taekwondo athletes were required for this study (at least 8 years of competitive experience and top 3 at national competitions), there were very few elite taekwondo athletes in this category. The subjects were all volunteers. Due to these factors, only 11 subjects were able to fully participate in this study. The sample size was also one of the limitations of this study. Furthermore, we suggest that additional conditions (i.e., control group, group training under safe conditions) could be added for comparison in further studies. During the 12 week period, we also instructed the athletes to maintain their habitual diet according to general dietary guidelines to minimize possible confounding factors.

## 5. Conclusions

Based on the above discussion, the following conclusions from this study can be summarized: (1) PIT shows better trends in body composition (arm and trunk muscle) and sport performances (acceleration and aerobic capacity) than DT does. (2) During the off-season, PIT is a useful training strategy for taekwondo athletes to maintain their sport performances, which is very practical for athletes and coaches to arrange adequate preparations for the next training period. During the spread of COVID-19 around the world, the PIT training model results will serve as a useful, safe guideline for training recommendations.

## Figures and Tables

**Table 1:** The PIT and DT training content.

	Competition Phase	12 Weeks of Personal Isolation Training or Detraining	
		PIT	DT
Training Content	Taekwondo-specific skill training, strength and conditioning training, stretching and flexibility training, and agility training	(1) Taekwondo-specific basic skills training: single kicking (2 h), combination kicking (2 h), intermittent kicking (1 h) (2) No-equipment strength and conditioning training: squat, jump, push-up, core muscle training (1.5 h) (3) Stretching and flexibility training (0.5 h)	No
Training Volume	14–15 h per week	7 h per week	No structured training

The training program is based on the traditional single-cycle annual training program as a scheduled training period [19].

**Table 2:** The body composition of PIT and DT.

Measurements	PIT		DT		PIT Δ%	DT Δ%
	T1	T2	T1	T2		
Weight (kg)	68.7 ± 3.4	70.1 ± 3.0	69.1 ± 0.5	70.5 ± 5.1	+2.3%	+1.8%
BMI (kg/m^2^)	22.2 ± 0.8	22.7 ± 0.7	23.1 ± 1.0	23.5 ± 1.1	+2.2%	+1.8%
LBM (kg)	58.6 ± 3.1	58.6 ± 3.3	56.9 ± 4.3	55.63 ± 4.6	+0.0%	−2.5%
Fat mass (%)	14.6 ± 2.3	16.5 ± 2.6 *	17.6 ± 2.2	21.1 ± 2.7 *	+16.5%	+20.5%
Muscle mass						
right arm (kg)	3.0 ± 0.2	3.1 ± 0.2	2.9 ± 0.3	2.8 ± 0.3 *	+2.9% #	−3.8%
left arm (kg)	3.0 ± 0.2	3.1 ± 0.2	2.9 ± 0.3	2.8 ± 0.3	+3.0%	−3.2%
trunk (kg)	24.3 ± 1.4	24.8 ± 1.3	23.9 ± 1.6	23.5 ± 1.8	+2.2% #	−1.9%
right leg (kg)	9.7 ± 0.6	9.6 ± 0.5	8.9 ± 0.7	8.8 ± 0.7	−1.0%	−1.1%
left leg (kg)	9.6 ± 0.6	9.5 ± 0.5	8.9 ± 0.7	8.7 ± 0.7	−1.2%	−1.5%

Values are expressed as mean ± S.E.M. PIT: personal isolation training, DT: detraining, Δ%: percent changes between pre- and post-test, T1: pre-test, T2: post-test, BMI: body mass index, LBM: lean body mass. * *p* < 0.05 vs. T1 under the same condition. # *p* < 0.05 vs. DT under the same condition.

## Data Availability

The data presented in this study are available upon request from the corresponding author.
